# Pre-Pandemic and Recent Oral and Medical Health Care Utilization among Young American Indian Children and Their Caregivers

**DOI:** 10.1007/s10900-024-01345-6

**Published:** 2024-03-11

**Authors:** Steven D. Barger, Christine Kirby, Heather Thomas, Carolyn Camplain, Sara Young, Gerlinda Morrison, Stephanie Hyeoma, Skyler J. Bordeaux, Chloe Horowitz, Julie A. Baldwin

**Affiliations:** 1 Department of Psychological Sciences, Northern Arizona University, Flagstaff, AZ USA; 2 Center for Health Equity Research, Northern Arizona University, Flagstaff, AZ USA; 3 Department of Dental Hygiene, Northern Arizona University, Flagstaff, AZ USA; 4 Department of Community and Population Health, Lehigh University, Bethlehem, PA USA; 5 Department of Health and Human Services, Hopi Tribe, Kykotsmovi, AZ USA

**Keywords:** American Indian or Alaska Native, Dental care for children, oral health, Child, Health care utilization, Preventive dentistry

## Abstract

Children from diverse ethnic groups are at significantly increased risk for dental caries. In particular, American Indian (AI) children have the highest incidence of detal caries of any ethnic group. The COVID-19 pandemic dramatically restricted health care access, including preventive oral health care. Given this context, it is unclear whether or not preventive oral health care for AI children has resumed since lockdown. To address this question, we surveyed adult AI caregivers (*N* = 152) of children aged 0–5 years, assessing recent (12-month) and pre-COVID (for caregivers of children aged 3–5 years) preventive oral and medical health services. We also examined medical health care access and utilization among caregivers. Among children aged 3–5 years old, both pre-pandemic and past year medical care utilization were generally high (80 and 90%, respectively) as was any oral health care utilization (64 & 78%, respectively). Oral health check-ups were more common over the last year (62%) compared to pre-COVID (44%). Recent health care utilization among children 1–5 years old in this sample were generally comparable to national estimates, except for higher reported preventive medical care (99% vs. 87.6%, respectively) and higher preventive oral care (96% vs. 59.6%, respectively). More caregivers reported delaying or foregoing needed health care due to COVID (28–38%) versus due to cost (8–17%). In this survey of AI caregivers, recent child preventive health care utilization was high, and changes in utilization following the lockdown phases of the pandemic were comparable for oral and medical health care.

## Introduction

Tooth decay is the most common chronic disease among U.S. children [1] and the largest caries burden occurs among racial and ethnic minorities [2, 3]. In particular, American Indian (AI) children have the largest caries burden of all racial/ethnic groups in the U.S., with rates 4 times higher than those of non-Hispanic white children. This burden is evident very early in life, with 1 of 6 one-year old AI children having early childhood caries [4].

Implementation of decay prevention programs by Indian Health Services and Tribal authorities [5] has likely contributed to the modest decrease in caries observed among AI children [4]. However, geographic isolation and a dearth of care providers in rural areas [6] may contribute to early childhood caries in these communities [7]. Additionally, the COVID-19 pandemic and associated lockdowns dramatically restricted health care access worldwide [8], and in the US preventive oral health care was unavailable for a time because many dental practices remained closed to all but emergency dental procedures [9]. Given this context of broadly delayed oral and medical health care services, it is unclear whether or not preventive oral health care for AI children has resumed since lockdown [9].

To address this health care utilization knowledge gap, we surveyed adult AI caregivers of children aged 0–5 years. We assessed evidence-based preventive oral health services for children using questions from national oral health surveillance instruments (National Survey of Children’s Health [10, 11]). Given the potential downstream impact of COVID-related preventive oral health care deficits [9], we also assessed children’s oral health quality of life [12]. This would reveal whether any untreated caries (perhaps due to COVID-19 restrictions) might present as difficulties in children’s daily activities (i.e., eating and/or sleeping).

Given challenges obtaining *any* health care during the pandemic, we also assessed medical health care access and utilization among children as well as their caregivers. This helps distinguish whether oral health care utilization patterns are distinct from, or aligned with, more general health care utilization. We asked caregivers to describe whether any unmet or delayed care over the last year (either oral or medical) was due to COVID-19 or to financial considerations. To provide a rough indication of changes in health care utilization due to COVID-19, we requested caregivers describe their child’s oral and medical health care both before the pandemic and in the last year (roughly December 2021-December 2022). This captures the extent to which preventive care resumed after widespread availability of vaccines and the termination of stay-at-home orders. Finally, to better understand previously identified barriers to obtaining care [13], such as geographic isolation [6, 7], we assessed transportation options, caregivers’ reasons for any missed oral or medical health care over the last 12 months, and travel time to care facilities.

Although we expected to see greater oral health care utilization prior to the pandemic, the overarching goal of the present study was to better understand the prevalence of and barriers to oral and medical health care utilization among an understudied population, AI caregivers and their young children.

## Methods

Our study used elements of a community based participatory (CBPR) approach. CBPR connects researchers with community partners to solve health and social issues through shared problem-solving and capacity building and honors the contributions of all partners [14]. We partnered with Community Health Representatives (CHRs) in each tribal community. CHRs have a role similar to Community Health Workers (CHWs) in other projects. In this study, CHRs were involved in recruiting participants, administering surveys, interviewing providers in the community, leading Community Advisory Board (CAB) meetings, and implementing a pilot intervention (described elsewhere). The CAB in each community helped tailor the educational materials for the study, connected the study team to local resources and participants, reviewed all study materials and provided formal and informal guidance on oral health practices and community health goals [15].

### Setting

We partnered with two Native Nations, one Southwestern tribe and one Plains tribe. Paper surveys were administered by CHRs on tribal lands by tribal members. Surveys were completed in person from July-December 2022. Participants were recruited in a variety of ways including at tribal cultural events, career fairs, a local tribal college, clinics, Head Start, WIC sites, community parks as well as via door to door and word of mouth. The study was not designed for tribe-specific estimates so results are pooled across sites.

### Eligibility

Persons were eligible to complete the survey if they were a) 18 years of age or older; 2) a member of one of the two tribes; and 3) either currently pregnant or a caregiver for at least one child 5 years of age or younger. If respondents had multiple children under 6 years of age, they were asked to refer to the oldest child < 6 years old when answering the survey. This age group was targeted because trials to improve pediatric oral health among Indigenous children are more successful when enrolling children from birth [16] rather than in elementary school [17] and because of the substantial caries burden evident among very young AI children [4]. The study was approved by the NAU IRB and tribal authorities and all respondents provided written informed consent to participate.

### Measures

#### Preventive Oral and Medical Health Care Services

We asked several questions regarding oral and medical health care, for both preventive care and for any other type of health care. We also assessed the specific type(s) of oral preventive services the child received (i.e., brushing instructions, teeth cleaning, fluoride treatment, etc.) [11]. These questions were taken from the National Survey of Children’s Health (NSCH) [10] and referred to the last 12 months. We asked these questions twice, with another version reworded to refer specifically before the COVID pandemic. We report pre-COVID data only for caregivers of children 3–5 years old at the time of the survey to ensure they were eligible for health care prior to the pandemic. We describe past 12 month preventive health care for children 1–5 years of age as caries burden in AI children is evident at 12 months [4]. We include U.S. prevalence estimates of these key utilization and prevention behaviors for comparison [18]. We report 2022 estimates for children 0–5 years old that combine racial and ethnic groups. We assessed caregiver-rated oral and general health status for the child, the former of which is associated with caries in AI children [19].

#### Oral Health Quality of Life

Poor oral health can affect the quality of life (QOL) of the child and the family and may be impacted by pandemic-related preventive oral health care deficits. To assess oral QOL caregivers answered three questions from the Early Childhood Oral Health Impact Scale [12]. Participants reported the extent to which the child had pain in the teeth, mouth or jaw. They were also asked whether the child had difficulty eating food or had trouble sleeping because of dental problems or dental treatments. Caregivers were instructed to consider the child’s entire life from birth until the present. These items were rated on a scale from *never*, *hardly ever*, *occasionally*, *often* or *very often*. Higher scores on the full scale are associated with more decayed and/or treated teeth [12] and with oral health behavior and caries in AI children [7, 19].

#### Caregiver Medical and Oral Healthcare Utilization and Barriers to Utilization

We asked caregivers about lifetime oral and medical care utilization as well as the last time they saw a doctor. We also asked about past year oral and medical care utilization, reasons for any missed appointments, usual transportation for health care visits as well as travel time to see oral and medical care providers. In addition, caregivers rated their overall and oral health and reported on their oral health behavior (brushing and flossing).

#### Caregiver Delayed or Missed Oral and Medical Care

To assess economic and COVID-related influences on recent health care utilization, we included two questions from the Access to Health Services instrument from the PHEN-X toolkit [20] (https://www.phenxtoolkit.org/protocols/view/270101). Caregivers were asked to report whether they had delayed or missed medical care because of the cost. We used the same wording to ask about delayed or missed oral health care due to cost and reworded these questions to determine whether care was delayed or missed due to COVID.

### Data Analysis

Data were analyzed using Stata 18 (Stata Corp, College Station, TX). Survey data were double entered and discrepancies resolved by research team members. Descriptive statistics are emphasized, but we provide statistical tests for pre-COVID and last 12-month health care utilization for children ages 3–5 as reported by their caregivers. McNemar’s test for dependent proportions was used to compare across the time periods, and a two-tailed *p*-value ≤ 0.05 was considered statistically significant.

## Results

### Caregiver Characteristics

Of the 197 surveys distributed, 24 respondents provided no data for a target child, 17 did not have a target child ≤ 5 years old and 4 were members of ineligible tribes, leaving 152 caregiver surveys (Table 1). Two respondents did not endorse American Indian race but both reported being a member of an eligible tribe. The majority of respondents were female (95%), employed (51%), and had some college education or higher (51%). Most caregivers reported health insurance via Medicaid (79%) and had access to a working vehicle (86%). However, half characterized their household income as *not enough to get by* or *barely enough to get by* (Table 1). Maintaining tribal identity was rated as very important by respondents and tribal language fluency was modest (Table 1). Caregivers’ general health status was reported as good or higher (78%) as was oral health status (68%). 49% of caregivers reported regularly brushing twice a day or more and 24% reported flossing daily.

**Table 1:** Caregiver Sociodemographic and Health Characteristics

Characteristic	N	%
Participants	152	
Age y, mean (SD)	32	9.0
Gender		
Female	145	95.0
Male	7	5.0
Hispanic ethnicity		
No	147	97.0
Yes	2	1.0
Don’t know	2	1.0
Missing	1	1.0
Race		
American Indian / Native American ^a^	150	99.0
Alaska Native	1	1.0
Asian	1	1.0
Native Hawaiian	2	1.0
Other Pacific Islander	1	1.0
Black/African American	1	1.0
White	4	3.0
Other	1	1.0
Education level		
< High school	21	14.0
High school diploma	49	32.0
Some college	68	45.0
College graduate or higher	9	6.0
Missing	5	3.0
Marital status		
Married/cohabiting	38	25.0
Divorced	9	6.0
Separated	9	6.0
Never married	51	34.0
Member of an unmarried couple	24	16.0
Other	13	9.0
Missing	8	5.0
Work force status ^b^		
Employed	78	51.0
Unemployed	30	20.0
Homemaker	13	9.0
Student	20	13.0
Retired	1	1.0
Missing	10	7.0
Importance of maintaining your Tribal identity ^c^		
A little	4	3.0
Somewhat	26	17.0
Very much	122	80.0
Tribal language fluency		
I don’t speak my tribal language	23	15.0
I speak it a little, but not very well	94	62.0
I speak it moderately well	26	17.0
I speak my tribal language very well	6	4.0
Missing	3	2.0
Persons in the household		
2	8	5.0
3	20	13.0
4	33	22.0
5	33	22.0
6	21	14.0
7	11	7.0
8	8	5.0
9 or more	13	9.0
Missing	5	3.0
Income adequacy		
Not enough to get by	16	11.0
Barely enough to get by	60	39.0
Sufficient to meet your needs	74	49.0
More than enough to meet your needs	1	1.0
Missing	1	1.0
Health care coverage		
None	6	4.0
A plan purchased through an employer or union	18	12.0
A plan that you or another family member buys on your own	1	1.0
Medicaid	120	79.0
Medicare	5	3.0
TRICARE (formerly CHAMPUS), VA, or Military	0	0.0
Alaska Native, Indian Health Service, Tribal Health Services	36	24.0
Some other coverage	2	1.0
Oral health care other than IHS?		
No	78	51.0
Yes	68	45.0
Don’t know	4	3.0
Missing	2	1.0
If yes, what kind of oral health care?		
A plan purchased through an employer or union	18	12.0
A plan that you or another family member buys on your own	1	1.0
Medicaid	44	29.0
Medicare ^d^	2	1.0
Don’t know	4	3.0
Missing	83	55.0
Do you have access to a working vehicle?		
No	22	14.0
Yes	130	86.0
Self-rated health		
Poor	0	0.0
Fair	30	20.0
Good	63	41.0
Very good	40	26.0
Excellent	18	12.0
Missing	1	1.0
Self-rated oral health		
Poor	13	9.0
Fair	34	22.0
Good	69	45.0
Very good	26	17.0
Excellent	9	6.0
Missing	1	1.0
How often do you brush your teeth? ^e^		
Do not brush my teeth	1	1.0
1 time per day	25	16.0
2 times per day	61	40.0
1 or 2 times per day	40	26.0
More than 2 times per day	13	9.0
Sporadically (I brush my teeth irregularly)	8	5.0
Missing	4	3.0
Do you use dental floss? ^e^		
No	8	5.0
Rarely	58	38.0
1–3 times per week	45	30.0
Daily	37	24.0
Missing	4	3.0

Values are N and percent unless otherwise noted. Age values exclude invalid responses

^a^ Two respondents selected “no” but affirmed membership in one of the two eligible tribes

^b^ Unable to work was coded as unemployed

^c^ “Not at all” was a response option but was not endorsed

^d^ Other insurance options were not endorsed

^e^ The response option “Not applicable (I have full dentures, upper and lower)” was not endorsed

### Pre-Pandemic Versus Past Year Child Health Care Utilization

Among children aged 3–5 years old, caregiver reports of child medical care, preventive medical and preventive oral health care were consistently higher over the last 12 months versus before the pandemic (Table 2). Both pre-pandemic and past year medical care utilization were generally high (80–90%) as was any oral health care utilization (64–78%). Oral health check-ups were more common over the last year (62%) compared to pre-COVID (44%), as were cleanings (52% vs. 33%), fluoride treatment (50% vs. 28%) and instructions on tooth brushing (29% vs. 14%) (Table 2).

**Table 2:** Child Medical and Oral Health Care Utilization and Health Status, Pre-COVID and Past Year, Children 3–5 Years Old

Variable	Pre-COVID (2019)		Past 12 months (2021–2022)	
	N	%	N	%
Any medical care				
No	19	19.0	10	10.0
Yes	81	80.0	90	90.0
Number of preventive medical visits				
0	3	4.0	1	1.0
1 or more	74	96.0	76	99.0
Any oral health care*				
No	36	36.0	22	22.0
Yes	65	64.0	79	78.0
Preventive oral health care**				
No	14	25.0	2	4.0
Yes	42	75.0	54	96.0
Type of preventive oral health care received ^a^				
Check up**				
No	57	56.0	38	38.0
Yes	44	44.0	63	62.0
Cleaning**				
No	68	67.0	48	48.0
Yes	33	33.0	53	52.0
Instruction on tooth brushing**				
No	87	86.0	72	71.0
Yes	14	14.0	29	29.0
Fluoride treatment**				
No	73	72.0	51	50.0
Yes	28	28.0	50	50.0
Sealant				
No	81	80.0	72	71.0
Yes	20	20.0	29	29.0
Don’t know	1	1.0	1	1.0

Note: Responses are limited to referent children 3 years of age or older at the time of the survey (through December, 2022). Only participants with non-missing values for both pre-COVID and last 12-month utilization are included to reflect pre-COVID and past 12-month comparisons

^a^ Some respondents reported receiving specific types of preventive care for their child despite not reporting a preventive oral health care visit

* *p* < 0.05 ** *p* < 0.01; McNemar’s exact test for dependent proportions

### Past Year Healthcare Utilization, Preventive Oral Health Care and Quality of Life

Among children aged 1–5, the prevalence of any medical care was similar to the past 12-month utilization among the 3-5-year-old subgroup, and lower for any oral health care (69% vs. 78%). Overall preventive oral health care was the same among children aged 1–5 (96%), and nominally lower when comparing different types of preventive oral health care (Table 3). Current child general health status was rated high, with 84% reporting *very good* or *excellent* health status. Oral health was rated relatively lower, with 60% rating their child’s oral health as *very good* or *excellent* (Table 3). Caregiver reports of their child’s oral quality of life were also positive, with caregivers reporting their child *never* or *hardly ever* experienced oral pain (82%), difficulty eating (87%) or difficulty sleeping (94%) due to oral health problems.

**Table 3:** Past 12-month Medical and Oral Health Care Utilization and Overall Health Status, Sample and National Estimates, Children Aged 1–5 Years

Variable	Study Sample (2021–2022)		2022 NSCH ^a^
	N	%	%
Any medical care ^b^			
No	15	11.0	9.7
Yes	123	89.0	90.3
Number of preventive medical visits ^b^			
0	1	0.8	12.4
1	41	31.5	87.6 (1 or more)
2 or more	88	67.7	
Any oral health care			
No	43	31.0	37.7
Yes, saw other oral health care provider	10	7.0	1.5
Yes, saw a dentist	85	62.0	59.8
Preventive oral health care			
No	5	4.7	40.4
Yes, 1 visit	58	54.2	59.6 (1 or more)
Yes, 2 or more visits	44	41.1	
Type of preventive oral health care received			
Check up			
No	62	45.0	44.7
Yes	76	55.0	55.3
Cleaning			
No	72	52.0	52.5
Yes	66	48.0	47.5
Instruction on tooth brushing			
No	102	74.0	67.4
Yes	36	26.0	32.6
Fluoride treatment			
No	77	56.0	70.1
Yes	61	44.0	29.9
Sealant			
No	105	76.0	95.1
Yes	33	24.0	4.9
Don’t know	3	2.0	
Child general health status (no time referent) ^c^			
Poor	1	0.9	0.8 (fair or poor)
Fair	0	0.0	
Good	17	15.0	6.0
Very good	33	29.2	93.2 (excellent or very good)
Excellent	62	54.9	
Child oral health status (no time referent) ^d^			
Poor	3	2.0	3.5 (fair or poor)
Fair	14	10.0	
Good	38	28.0	10.7
Very good	53	38.0	85.7 (excellent or very good)
Excellent	30	22.0	
Child oral quality of life (birth to present)			Not available
Pain in mouth, teeth or jaw			
Never	67	49.0	—
Hardly ever	45	33.0	—
Occasionally	15	11.0	—
Often	5	4.0	—
Very often	1	1.0	—
Don’t know	5	4.0	—
Difficulty eating due to dental problems			
Never	94	68.0	—
Hardly ever	26	19.0	—
Occasionally	14	10.0	—
Often	1	1.0	—
Very often	2	1.0	—
Don’t know	1	1.0	—
Difficulty sleeping due to dental problems			
Never	110	80.0	—
Hardly ever	20	14.0	—
Occasionally	6	4.0	—
Often	1	1.0	—
Very often	1	1.0	—

NSCH, National Survey of Children’s Health [18]. Quality of life items are from the Early Childhood Oral Health Impact Scale [12]. Missing data are excluded for prevalence estimates to better approximate calculations in the NSCH

^a^ Percentages were obtained using interactive data queries at www.childhealthdata.org. Examining American Indian respondents separately in the NSCH is not possible due to the small number of American Indian respondents. Median sample size for the NSCH estimates is 7,190, with the lowest number of responses for sealants (*N* = 315)

^b^ NSCH age range is 0–5 years for this outcome

^c^ The question in the present study was “Would you say that in general, your child’s health is…” whereas in the NSCH it was “In general, how would you describe this child’s health?”

^d^ The question in the present study was “Would you say that in general, the health of your child’s teeth and mouth are…” whereas for the NSCH it was worded “How would you describe the condition of the child’s teeth?”

Health care utilization and preventive oral health care in children 1–5 years old were generally comparable to national estimates for children aged 5 and under [18], except for more frequent preventive medical care (99% vs. 87.6%, respectively), preventive oral care (96% vs. 59.6%, respectively) and dental sealant use (24% vs. 4.9%, respectively). Sample-specific estimates are more sensitive to excluding missing data compared to national estimates, so these comparisons should be interpreted cautiously. The proportion of caregivers rating their child’s general (84% vs. 93.2%) and oral health (60% vs. 85.7%, respectively) as *very good or excellent* were lower in our sample versus national estimates (Table 3).

### Caregiver Medical and Oral Health Care and Barriers to Utilization

80% of caregivers reported ever seeing a medical provider and 91% of caregivers reported ever seeing an oral health care provider (Table 4). The majority (77%) of caregivers attended scheduled medical appointments *most* or *every* time (Table 4) but this was lower for oral health appointments (51%). The most common reasons a medical/dental appointment was missed were not being able to take off work or school, lacking transportation, family commitments, and forgetting (Table 4). Missing appointments because of COVID infection or fear of COVID infection was reported by 3–6% of respondents. Respondents typically traveled to their medical and dental appointments using their own vehicle (66%) and travel time to medical and dental appointments was comparable (Table 4).

**Table 4:** Caregiver Medical and Oral Health Care and Barriers to Utilization

	Type of Care			
	Medical care/doctor		Dental care/dentist	
	N	%	N	%
Have you ever been to the doctor/dentist?				
No	29	19.0	14	9.0
Yes	121	80.0	138	91.0
Missing	2	1.0		
Months since seeing doctor				
6 months or less	91	60.0	N/A	N/A
7–12 months	12	8.0		
13–23 months	5	3.0		
24 or more months	7	5.0		
Missing	37	24.0		
How often have you been able to attend scheduled medical/dental appointments in the last 12 months?				
I had no scheduled medical/dental appointments in the last 12 months	9	6.0	31	20.0
Attended few of my appointments	8	5.0	9	6.0
Attended some appointments	13	9.0	17	11.0
Attended most appointments	60	39.0	41	27.0
Attended every appointment	58	38.0	37	24.0
Missing	4	3.0	17	11.0
What are the reasons you have not been able to attend a scheduled medical/dental appointment?				
I forgot	16	11.0	25	16.0
I could not take off work or school	29	19.0	26	17.0
I had anxiety about the appointment that prevented me from going	6	4.0	14	9.0
I had other family commitments	17	11.0	15	10.0
The person I was depending on to take me couldn’t do it	10	7.0	15	10.0
I was too sick to attend	3	2.0	6	4.0
I did not have transportation	28	18.0	26	17.0
I was afraid I would not be able to pay for the visit	2	1.0	1	1.0
I realized my insurance was not up to date or would not cover the visit	0	0.0	3	2.0
I did not have time to go	10	7.0	11	7.0
Health care provider canceled the appointment	9	6.0	8	5.0
Fearful about contracting COVID-19 at the appointment	4	3.0	9	6.0
I was infected with COVID-19 or isolating	4	3.0	7	5.0
Other	0	0.0	0	0.0
Have you ever missed a medical appointment because you didn’t have transportation to get there?				
No	76	50.0	N/A	N/A
Yes	73	48.0		
Missing	3	2.0		
How do you usually get to your medical/dental appointments?				
Your own vehicle	100	66.0	101	66.0
Borrow a vehicle	19	12.0	29	19.0
Friend or family member	33	22.0	42	28.0
Public transportation	5	3.0	8	5.0
Taxi/ride share	1	1.0	1	1.0
Non-emergency medical transportation	3	2.0	8	5.0
Tribal offered transportation	0	0.0	3	2.0
Community health representative	1	1.0	1	1.0
Walking	3	2.0	2	1.0
Hitchhiking (catching a ride)	1	1.0	2	1.0
Other	0	0.0	0	0.0
I don’t go to medical/dental appointments	0	0.0	0	0.0
Travel time to medical office/dentist office				
0–15 min	35	23.0	42	28.0
16–30 min	40	26.0	35	23.0
31–60 min	36	24.0	32	21.0
61–90 min (> 1-1.5 h)	21	14.0	18	12.0
91–120 min (> 1.5-2 h)	11	7.0	8	5.0
More than 120 min (> 2 h)	3	2.0	7	5.0
Don’t know/missing	6	4.0	10	7.0

### Financial and COVID-related Barriers to Health Care Utilization

Over the past 12 months, 8% of caregivers reported delaying medical care due to cost and 17% reported delaying oral health care due to cost. Similar prevalence was observed for caregivers foregoing needed medical and oral health care due to cost (10% and 17%, respectively) (Table 5). Delayed or missed medical and oral health care over the last 12 months was more likely due to concerns about COVID (ranging from 28 to 38% of respondents).

**Table 5:** Caregiver Delayed or Missed Medical and Oral Health Care, Past 12 Months

	Reason			
	Cost		COVID-19	
	N	%	N	%
During the past 12 months, have you DELAYED getting medical care because of ______?				
No	134	88.0	93	61.0
Yes	12	8.0	58	38.0
Don’t know/missing	6	4.0	1	1.0
During the past 12 months, was there any time when you needed medical care, but DID NOT GET IT because of ______?				
No	134	88.0	101	66.0
Yes	15	10.0	43	28.0
Don’t know/missing	3	2.0	8	5.0
During the past 12 months, have you DELAYED getting dental care because of ______?				
No	123	81.0	95	62.0
Yes	26	17.0	55	36.0
Don’t know/missing	3	2.0	2	1.0
During the past 12 months, was there any time when you needed dental care, but DID NOT GET IT because of ______?				
No	121	80.0	102	67.0
Yes	26	17.0	47	31.0
Don’t know/missing	5	3.0	3	2.0

## Discussion

In this study of American Indian caregivers and their young children, we found that children’s oral health care utilization was higher in the last 12 months compared to before the pandemic, and that the overall prevalence of preventive oral health care was higher than national estimates. The latter could in part be due to a substantial number of missing responses (22%) and should be interpreted accordingly. Although medical care for children was generally more common than oral health care, changes from pre-pandemic to past year utilization were comparable for both medical and oral health care, indicating alignment of both types of health care during this period. The apparent increases in utilization could be a function of “catching up” with care after lockdowns, a greater accumulation of health care utilization because the child is getting older, or some combination of these and other factors. In any case, these data are consistent with a general recovery of health care among children of these AI caregivers relative to the start of the pandemic.

Oral health quality of life among these young children is generally good, suggesting that pandemic-related barriers to healthcare may not have adversely affected their oral health. Although we did not measure caries, higher oral health quality of life is associated with fewer caries in AI children [7, 12, 19] so these data are encouraging. Conversely, reported general and oral health status ratings for these children are lower compared to national data, disparities that mirror findings in adults [21] and merit ongoing public health attention. Although we feel the 2022 NSCH are the best comparison data available, apparent discrepancies (or alignments) of those data with the present study should be interpreted cautiously, given the NSCH data is a probability sample and aggregates heterogeneous racial and ethnic groups.

Rural settings and a dearth of providers are hypothesized barriers to health care access among AI populations [22]. Although most respondents reported having access to a working vehicle, transportation was a commonly mentioned barrier to attending scheduled medical and oral health care appointments [13], as were conflicts with work or school. Notably, insurance coverage was rarely reported as a barrier to receiving care and 79% of the sample reported having Medicaid insurance. Financial concerns were not reported as strong determinants of missing or delaying past year health care among caregivers, whereas COVID-related concerns were endorsed by a quarter to over a third of respondents. Most importantly, 28–31% of respondents reported not getting needed medical or oral health care because of COVID. These characterizations of barriers to health care among rural AI populations can inform community and tribal efforts to improve health care access.

### Strengths and Limitations

The present study was a collaborative effort with tribal communities, providing a broad assessment of medical and oral health care among AI caregivers and their young children. We assessed barriers to seeking and obtaining health care and estimated recent preventive oral health care among younger children using assessments from national survey instruments. These data provide an important snapshot of health care utilization among community members of Southwestern and Plains AI tribes, but may not be generalizable to tribes in other geographic locations in the U.S.

The study is subject to a number of limitations. We relied on caregivers’ retrospective reports of health care utilization. However, retrospective reporting may have been difficult for some participants, especially given the traumatic events during the COVID pandemic, with so much loss of lives and illness in tribal communities. The reported increases in recent health care utilization compared to pre-COVID could be due to healthier persons participating in the survey, who in turn may be more likely to re-initiate health care after lockdowns. Those with access to transportation may also have been more likely to participate in the study. There may also be influences of the child’s age in the apparent increase in utilization, in that older children may be more likely to have seen an oral health care provider versus younger children. This is consistent with the slightly lower percentage (69%) of 1-5-year olds having any oral health care versus 3-5-year olds (78%). These differences did not translate into substantially lower percentages of the different types of preventive oral health care across the group including younger (1-5-year-olds) versus older (3-5-year-olds) children.

## Conclusions

This study represents one of the first to examine oral health care utilization among AI children and their caregivers as reported before and towards the end of the COVID pandemic. The results were somewhat surprising in seeing an uptake of preventive oral health behaviors in the last months of the pandemic and also seeing rates of reported behaviors very similar to a national study of children the same age. Our team expected to see more reported challenges in accessing and receiving oral health care toward the tail end of the pandemic as compared to before the pandemic, and as compared to a national sample. Whether there might be some selection bias in our sample (i.e., a more health-conscious group of participants, those with more access to care, and/or those less affected by the pandemic) or other factors which could account for these observations, this study represents an interesting snapshot in time and has implications for future research. Specifically, in future studies, it would be beneficial to look more carefully at what characterizes those who reported better oral health care utilization and preventive care versus those who did not. What factors might have made a difference, and can these be factors be considered in developing and implementing future oral health care interventions with AI children and their caregivers? Given the persistent and harmful disparities in ECC among AI children, it is imperative that community-driven interventions address numerous barriers to oral health care – whether at the individual and family levels and/or community and health care system levels. This study represents a first attempt to elucidate some of these questions and provides a foundation upon which to build future work.

## Data Availability

As per Tribal agreements the data are not available to outside investigators.
